# Integrated Action of Autophagy and Adipose Tissue Triglyceride Lipase Ameliorates Diet-Induced Hepatic Steatosis in Liver-Specific PLIN2 Knockout Mice

**DOI:** 10.3390/cells10051016

**Published:** 2021-04-25

**Authors:** John D. Griffin, Eloy Bejarano, Xiang-Dong Wang, Andrew S. Greenberg

**Affiliations:** 1 Obesity and Metabolism Laboratory, Jean Mayer USDA Human Nutrition Research Center on Aging, Tufts University, Boston, MA 02111, USA; John.Griffin@Tufts.edu; 2 Laboratory for Nutrition and Vision Research, Jean Mayer USDA Human Nutrition Research Center on Aging, Tufts University, Boston, MA 02111, USA; Eloy.Bejarano@Tufts.edu; 3 School of Health Sciences, Universidad CEU Cardenal Herrera, 46001 Valencia, Spain; 4 Laboratory for Nutrition and Cancer Biology, Jean Mayer USDA Human Nutrition Research Center on Aging, Tufts University, Boston, MA 02111, USA; Xiang-dong.Wang@tufts.edu

**Keywords:** perilipin, lipid droplet, autophagy, adipose tissue triglyceride lipase, non-alcoholic fatty liver disease

## Abstract

An imbalance in the storage and breakdown of hepatic lipid droplet (LD) triglyceride (TAG) leads to hepatic steatosis, a defining feature of non-alcoholic fatty liver disease (NAFLD). The two primary cellular pathways regulating hepatic TAG catabolism are lipolysis, initiated by adipose triglyceride lipase (ATGL), and lipophagy. Each of these processes requires access to the LD surface to initiate LD TAG catabolism. Ablation of perilipin 2 (PLIN2), the most abundant lipid droplet-associated protein in steatotic liver, protects mice from diet-induced NAFLD. However, the mechanisms underlaying this protection are unclear. We tested the contributions of ATGL and lipophagy mediated lipolysis to reduced hepatic TAG in mice with liver-specific PLIN2 deficiency (PLIN2^LKO^) fed a Western-type diet for 12 weeks. We observed enhanced autophagy in the absence of PLIN2, as determined by ex vivo p62 flux, as well as increased p62- and LC3-positive autophagic vesicles in PLIN2^LKO^ livers and isolated primary hepatocytes. Increased levels of autophagy correlated with significant increases in cellular fatty acid (FA) oxidation in PLIN2^LKO^ hepatocytes. We observed that inhibition of either autophagy or ATGL blunted the increased FA oxidation in PLIN2^LKO^ hepatocytes. Additionally, combined inhibition of ATGL and autophagy reduced FA oxidation to the same extent as treatment with either inhibitor alone. In sum, these studies show that protection against NAFLD in the absence of hepatic PLIN2 is driven by the integrated actions of both ATGL and lipophagy.

## 1. Introduction

Hepatic triglyceride (TAG) is stored within cytoplasmic lipid droplets (LD), an organelle responsible for sequestering neutral lipid and regulating its release [1,2]. An imbalance in the storage and release of hepatic LD TAG leads to the excessive accumulation of lipid within the liver, known as hepatic steatosis. Hepatic steatosis is a defining feature of non-alcoholic fatty liver disease (NAFLD), which is strongly associated with obesity and aging, and predisposes to the development of diabetes, cardiovascular disease, steatohepatitis, cirrhosis, and liver cancer [3,4,5,6,7,8]. 

The two major pathways regulating LD TAG catabolism are lipolysis and autophagy mediated hydrolysis of LD TAG, a process termed “lipophagy” [9]. Lipolysis is thought to be initiated by association of the cytosolic adipose triglyceride lipase (ATGL) with the LD surface, where it catalyzes the release of fatty acids (FA) from stored TAG, generating diacylglycerol and FA. The resulting DAG is ultimately hydrolyzed to its two components, FA and glycerol, by the sequential actions of hormone-sensitive lipase and monoacylglycerol lipase. Lipophagy also occurs at the surface of the LD, where a network of proteins including Rab family members and autophagy-related family members facilitate the engulfment of LD lipid in specialized degradative vesicles, called autophagosomes. Breakdown of LD TAG contained within the autophagosome occurs when these lipid containing vesicles fuse with lysosomes, exposing their contents to the hydrolase, lysosomal acid lipase. While the enzymatic machinery underpinning these processes is distinct, both lipolysis and autophagy require access to the lipid droplet surface for initiation. 

Access to the lipid droplet surface is tightly controlled by perilipins (PLIN), a class of lipid droplet-associated proteins which regulate LD TAG storage and release [1]. PLIN2 is the most abundant member of the PLIN protein family in steatotic livers of mice and humans and its expression is strongly correlated with the severity of steatosis [10,11,12,13]. Furthermore, mouse models of PLIN2 deficiency have consistently demonstrated a reduction in hepatic TAG, highlighting the importance of PLIN2 in the development of obesity-associated hepatic steatosis [11,14,15,16,17,18,19,20]. 

PLIN2 is believed to regulate LD TAG levels by acting as a barrier, blocking lipase access to neutral lipids contained within LD [21]. In support of this view, overexpression of PLIN2 increases TAG accumulation by excluding ATGL from the LD surface, thereby reducing rates of lipolysis [22]. In addition to regulation of lipolysis, PLIN2 has also been implicated in the regulation of hepatic lipophagy, with increased rates of autophagy observed in livers of whole-body deficient PLIN2 mice [23]. In sum, these data suggest that increases in both lipolysis and lipophagy may contribute to the reductions in hepatic TAG observed with PLIN2 deficiency. While lipolysis and lipophagy were traditionally thought of as distinct metabolic pathways, emerging evidence suggests that these processes function in tandem and that cooperation between these two pathways is essential for complete LD hydrolysis [24,25]. To date the integrated actions of these processes has not been explored in PLIN2-deficient models.

Despite consistent reductions in the severity of hepatic steatosis following PLIN2 ablation, the influence of hepatic PLIN2 loss on the development NAFLD comorbidities such as obesity and insulin resistance during disease progression remains unresolved. Following short-term, 12 week high-fat diet treatment, increased insulin sensitivity and reduced adiposity are observed in mice with whole-body knockout or antisense-oligonucleotide knockdown of PLIN2 [11,14,15,16,20]. Conversely, obesity and glucose intolerance develop in mice with hepatic-specific deficiency of PLIN2 after 30 week Western-type diet feeding. These data suggest that hepatic-specific deficiency of PLIN2 may only protect from obesity and insulin resistance early in the progression of NAFLD, although this has yet to be explored. 

In the present study, liver-specific PLIN2 knockout (PLIN2^LKO^) mice were fed a Western-type diet for 12 weeks to (1) evaluate the contributions of lipolysis and lipophagy to reductions in hepatic TAG following PLIN2 deficiency, as well as (2) to delineate the effect of hepatic PLIN2 loss, specifically, on the development of obesity and insulin resistance early in the progression of NAFLD. Our study reveals that hepatic-specific loss of PLIN2 reduces hepatic TAG but does not reduce adiposity or improve insulin resistance in mice fed a WTD for 12 weeks. We find that liver-specific deficiency of PLIN2 increases autophagy in vivo and show that the integration action of lipophagy and ATGL-mediated lipolysis is necessary for increased FA oxidation following loss of PLIN2. 

## 2. Materials and Methods

### 2.1. Animal Care

All experiments were conducted at the Jean Mayer U.S. Department of Agriculture Human Nutrition Research Center on Aging in accordance with guidelines and regulations approved by the Institutional Animal Care and Use Committee of Tufts University. All mice starting at weaning were fed purified, chow diet (2016S, Teklad). Individually housed male PLIN2^fl/fl^ and PLIN2^LKO^ littermates were randomized into dietary treatment groups at 8 or 16 weeks of age where indicated. Animals received either an experimental, Western-type diet (WTD, Research Diets D15102305) containing 17% protein, 44% fat, and 39% carbohydrate by energy (kcal), or a micronutrient-matched low-fat control diet (LFD, Research Diets D15102307) containing 17% protein, 10% fat, and 73% carbohydrate by energy. The macronutrient composition of the WTD was modeled after a high-fat, high-sucrose diet with a demonstrated ability to induce hepatic steatosis after 12 weeks of feeding (diet composition available in Table S1 and S2) [26]. Following 12 weeks of dietary treatment animals were fasted for 6 h and euthanized via exsanguination under isoflurane anesthesia. Tissues were collected, snap frozen in liquid nitrogen, and stored at −80 °C. Blood was collected in EDTA coated tubes, spun at 2000× *g* for 20 min at 4 °C, and plasma fraction was collected and stored at −80 °C.

### 2.2. In Vivo Phenotyping

Body weight and body composition were analyzed by magnetic resonance imaging (EchoMRI-700) at baseline and 2, 4, 8, and 12 weeks following start of WTD feeding. Insulin tolerance tests (ITTs) were performed after 10 weeks of diet feeding in mice started on diet at 16 weeks of age. For ITTs animals were fasted for 6 h prior to intraperitoneal injection of 0.75 IU/kg insulin dissolved in sterile saline. Blood glucose was monitored from the tail vein via glucometer (OneTouch Ultra) at baseline (time 0) and 15, 30, 45, 60, 90, and 120 min following injection. Glucose tolerance tests (GTTs) were performed after 10 weeks of diet feeding in mice started on diet at 16 weeks of age. GTTs were performed similarly to ITTs with animals treated with 1.5 g/kg glucose dissolved in sterile saline delivered via intraperitoneal injection. As an estimate of insulin resistance, the Homeostasis Model Assessment of Insulin Resistance (HOMA-IR) was calculated as previously described using fasting insulin and blood glucose collected at time of sacrifice [16]. 

### 2.3. Histology

Following sacrifice, liver samples were fixed in either 4% zinc-buffered formaldehyde (Z-Fix) at room temperature for 48 h and then stored in 70% alcohol at 4 °C or frozen optimal cutting temperature solution and stored at −80 °C. Fixed samples were embedded in paraffin, sectioned, and stained with hematoxylin and eosin (H&E) and picrosirius red. Images were collected using an Olympus BX51-P polarizing light microscope with a ×40 objective. For histopathological analysis, sections were examined under light microscopy by one independent investigator who was blinded to the treatment groups. A ZEISS microscope with a PixeLINK USB 2.0 (PL-B623CU) digital Camera and PixeLINK μScope Microscopy Software was used for quantification and image capture for histopathological analyses. For each animal, 20 fields at a magnification of 10× were examined. Hepatic steatosis was graded based on the percentage of the liver section that was occupied by fat vacuoles (both macro- and micro-vesicular fat accumulation) at 100× magnification in 20 fields (grade 0 = < 5%; grade 1 = 5 to 25%; grade 2 = 26 to 50%; grade 3 = 51 to 75%; grade 4 = 76% to 100%). Inflammatory foci ((clusters of the recruited immune cells) and ballooning degeneration of hepatocytes were also examined.

### 2.4. Plasma Biochemistry

All plasma measures were obtained from samples collected at time of sacrifice as described above. Plasma insulin was determined via ELISA (Ultra-Sensitive Mouse Insulin ELISA, Crystal Chem, Zaandam, Netherlands). Triglyceride (OSR6133, Beckman Coulter, Galway, Ireland), cholesterol (OSR6116, Beckman Coulter), HDL (OSR6195, Beckman Coulter), β-hydroxybutyrate (LiquiColor, StanBio, Boerne, TX, USA), and non-esterified fatty acids (NEFA-HR (2), Wako Diagnostics) were determined calorimetrically.

### 2.5. Hepatic Lipids

An amount of 50–75 mg of frozen, pulverized liver was mechanically homogenized (TissueLyser II, Qiagen, Milan, Italy) in ice-cold 2:1 chloroform-methanol and lipids were extracted following the methods of Folch et al. [27]. Lipid extract was dried under N_2_ and solubilized in 1.5% Triton-X in phosphate-buffered saline. The protein fraction was isolated, dried under N_2_, and digested in 1 N NaOH for 96 h at 4 °C. Triglyceride and hepatic non-esterified fatty acids (NEFA) were determined calorimetrically as described in Plasma Biochemistry above. Hepatic lipids were normalized per mg protein, as determined by BCA Assay (BCA Protein Assay Kit, Pierce).

### 2.6. Primary Hepatocyte Isolation and Measurement of Fatty Acid Oxidation

Mouse primary hepatocytes were isolated from chow-fed, 3–9-month-old PLIN2^fl/fl^ and PLIN2^LKO^ animals following the collagenase perfusion method as previously described (18). Viability of isolated hepatocytes was routinely assessed via trypan blue exclusion and only isolations yielding 90% or greater viable cells were used for study. Following 4 h of plating, hepatocytes were incubated overnight in serum-free M199 media (23 mM HEPES, 26 mM sodium bicarbonate, 10 nM dexamethasone, 10 nM insulin, and 11 mM glucose) containing antibiotic/antimycotics. The following morning hepatocytes were pulsed for 2 h with serum-free M199 media containing 500 μM oleic acid and 0.1 µCi/mL ^14^C oleate. Following pulse cells were washed in PBS and chased in serum-free M199 media lacking insulin for 6 h. At the end of the experiment, media were collected and FA oxidation, as defined by the incorporation of ^14^C-oleate into CO_2_ and acid-soluble metabolites, was determined as previously described [19]. Data were normalized per mg protein, as determined by harvesting cells at the termination of the experiment. 

### 2.7. Autophagy Analysis

For ex vivo analysis, livers from mice fed a 60% high-fat diet (HFD, Research Diets D12492) for 12 weeks were harvested, washed briefly in PBS, minced on ice, and then placed in pre-warmed 12-well plates containing 37 °C M199 media supplemented with 20 mM NH_4_Cl and 200 µM leupeptin. Following a 2 h incubation at 37 °C, well contents were collected, and tissue was pelleted via centrifugation. The supernatant was discarded, and pellet washed once in ice-cold PBS. Tissue was then homogenized as described below and Western blotting against the autophagic adaptor p62 was used to evaluate p62-autophagic flux. Immunostaining of endogenous LC3 and p62 was used to evaluate autophagic activity. PLIN2^fl/fl^ and PLIN2^LKO^ hepatocytes were incubated with complete medium, medium with 100 µM chloroquine (CQ), 30 µM ATGL-specific inhibitor ATGListatin (ATGLi) (Xcess Biosciences, San Diego, CA, USA), or medium containing both CQ and ATGLi treatments for 4 or 6 h (as specified in figure legends) and fixed in cold methanol. Hepatic tissues were embedded in OCT for immunohistochemistry and 10 μm cryosections were obtained, dried overnight, and stored at −80 °C until staining. Indirect immunofluorescence was performed following conventional procedures, as previously described [28,29]. ImageJ was used to calculate area occupied by autophagic vesicles per total cellular area.

### 2.8. Western Blot Analysis

Protein extract was prepared by mechanical homogenization of 50 mg of frozen, pulverized liver tissue in ice-cold TNET Buffer (50 mM Tris-HCl, 150 mM NaCl, 2 mM EDTA, 1% Triton X-100, and 0.5% cholate) containing protease inhibitors (A32965, Thermo Scientific, Waltham, MA, USA). Protein concentration of extract was determined by BCA Assay as described above. Prior to gel loading, extracts were denatured by boiling at 95 °C for 5 min in an equal volume of 2× Laemelli Buffer (S3401-1VL, Sigma, St. Louis, MO, USA). An amount of 25–50 μg of protein was resolved in 10% polyacrylamide gels (Mini Protean TGX, BioRad, Hercules, CA, USA) and subsequently transferred to 0.45 μm nitrocellulose membranes (BioRad) at 300 mA for 2 h at 4 °C. Following transfer, membranes were blocked in 5% bovine serum albumin in phosphate-buffered saline containing 0.1% Tween-20 (PBST), washed three times in PBST, and incubated with primary antibody overnight. Blots were probed for PLIN2 (20R-AP002, Fitzgerald), PLIN3 (10694-1-AP, Proteintech, Rosemont, IL, USA), PLIN4 (rabbit anti-serum provided by Dr. Nathan Wollins), PLIN5 (GP44, Progen, Heidelberg, Germany) and GAPDH (14C10, Cell Signaling) using anti-rabbit secondary antibody (7074S, Cell Signaling, Frankfurt/Main, Germany). Proteins were detected by incubation with chemiluminescent reagent (34080, Thermo Scientific) and developed using x-ray film (34090, Thermo Scientific). Protein abundance was quantified densitometrically using ImageJ software and normalized to GAPDH. 

### 2.9. Quantitative Real-Time PCR

An amount of 30–50 mg of pulverized liver tissue was mechanically homogenized in TRIzol (Invitrogen, Carlsbad, CA, USA) and total RNA was extracted with RNeasy Mini columns (74106, Qiagen) as per the manufacturer’s instructions. RNA concentration and purity of extracts were determined using a Nanodrop 100 spectrophotometer. cDNA was generated from 2 µg RNA using a High-Capacity cDNA Reverse Transcription Kit (Applied Biosystems, Foster City, CA, USA). Quantitative real-time PCR (qPCR) was performed using SYBR Green (Applied Biosystems) on an Applied Biosystems 7300 Real-Time PCR System. Relative expression was quantified using the 2-ΔΔCt method [20]. Primer sequences are listed in Table S3. 

### 2.10. Statistical Analysis

Values are expressed as the means and standard errors of the mean. Student’s t-test and two-way analysis of variance (ANOVA) followed by Tukey’s HSD post hoc testing was performed using R (Version 3.5.0). Values of *p* ≤ 0.05 were considered significant.

## 3. Results

### 3.1. Generation of PLIN2^LKO^ Mice

**Figure 1:**
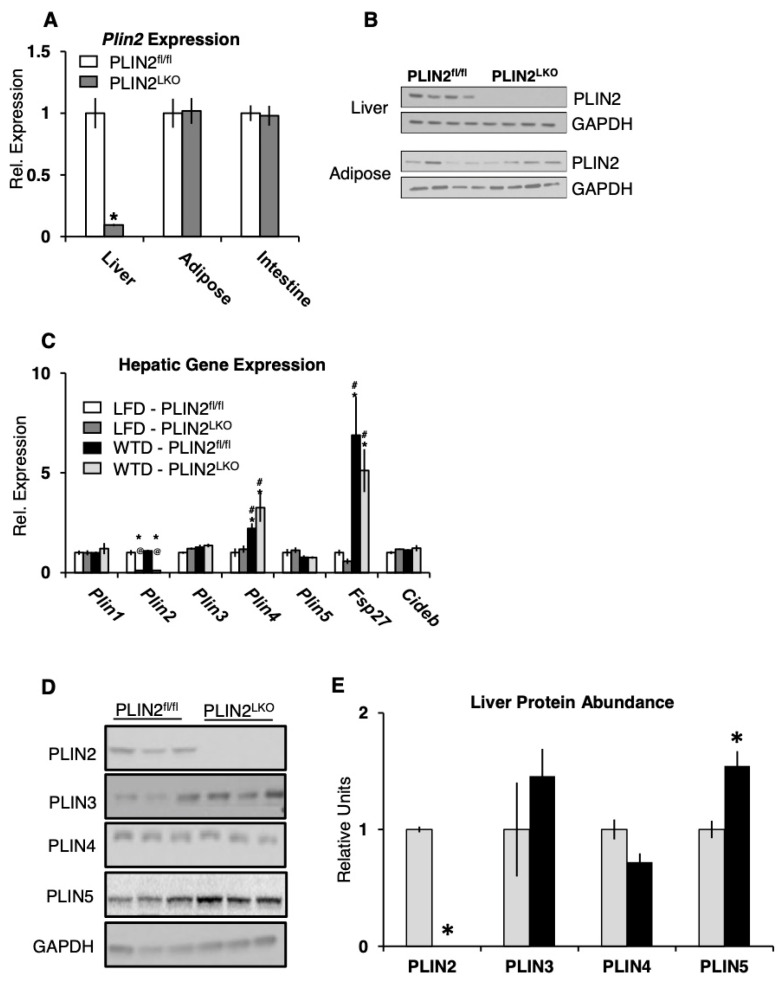
PLIN5 protein abundance is significantly increased by liver-specific loss of PLIN2. (**A**) Gene expression of PLIN2 in tissues from PLIN2^fl/fl^ (white) and PLIN2^LKO^ (grey) mice fed low-fat diet for 12 weeks. *n* = 8–10/group. (**B**) Representative blots of PLIN2 protein expression in liver and adipose from mice fed a Western-type diet for 12 weeks. (**C**) Gene expression in liver of 20-week-old mice following 12 weeks of LFD or WTD treatment. *n* = 8–13/group. Data are presented as the mean ± SEM. * *p* ≤ 0.05 vs. LFD–PLIN2^fl/fl^, # *p* ≤ 0.05 vs. LFD–PLIN2^LKO^, and @ *p* ≤ 0.05 vs. WTD–PLIN2^fl/fl^, as determined by two-way ANOVA and Tukey’s post hoc analysis. (**D**) Representative blots and (**E**) densitometric analysis of PLIN protein abundance in liver following 12 weeks of WTD treatment. Data are presented as the mean ± SEM. * *p* ≤ 0.05 vs. WTD–PLIN2^fl/fl^, as determined by student’s *t*-test.

Mice with liver-specific deficiency of PLIN2 (PLIN2^LKO^) were generated by mating mice harboring loxP segments flanking exon 5 of the *Plin2* gene (PLIN2^fl/fl^) [11] to mice carrying an albumin-Cre transgene. Western blot and qPCR analysis confirmed reduced protein and gene expression in livers PLIN2^LKO^ mice (Figure 1A,B). Consistent with tissue specificity of PLIN2 deficiency, PLIN2 protein levels were not changed in small-intestine and gonadal white adipose (gWAT) of PLIN2^LKO^ mice (Figure 1A,B). For our studies, 8-week-old PLIN2^fl/fl^ and PLIN2^LKO^ mice were randomized to either a low-fat (LFD) or a high-fat, Western-type diet (WTD), which has been demonstrated to induce hepatic steatosis by 12 weeks (Table S1) [26].

To determine whether hepatic PLIN2 deficiency altered the expression of other LD-associated proteins, we first performed RT-PCR analysis, which demonstrated no difference in *Plin1*, *Plin3*, *Plin4*, *Plin5*, *Fsp27* and *Cideb* mRNA expression between the two lines of mice during both LFD and WTD feeding (Figure 1C). Since protein expression of the perilipin proteins is strongly regulated post-translationally by level of LD TAG [30], we performed Western blot analysis to determine changes in PLIN isoform abundance. We observed no difference in protein abundance of PLIN3 and PLIN4 between the two lines of mice following WTD feeding (Figure 1D,E). Surprisingly, the protein abundance of PLIN5 was increased by 50% in PLIN2^LKO^ livers. In summary, these results suggest that after 12 weeks of WTD feeding, liver-specific loss of PLIN2 does not affect expression of other LD proteins except for PLIN5, an isoform implicated in the regulation of lipid storage, oxidation and autophagy [31,32,33].

### 3.2. PLIN2^LKO^ Mice Are Not Protected from Western-Type Diet-Induced Weight Gain and Insulin Resistance

**Figure 2:**
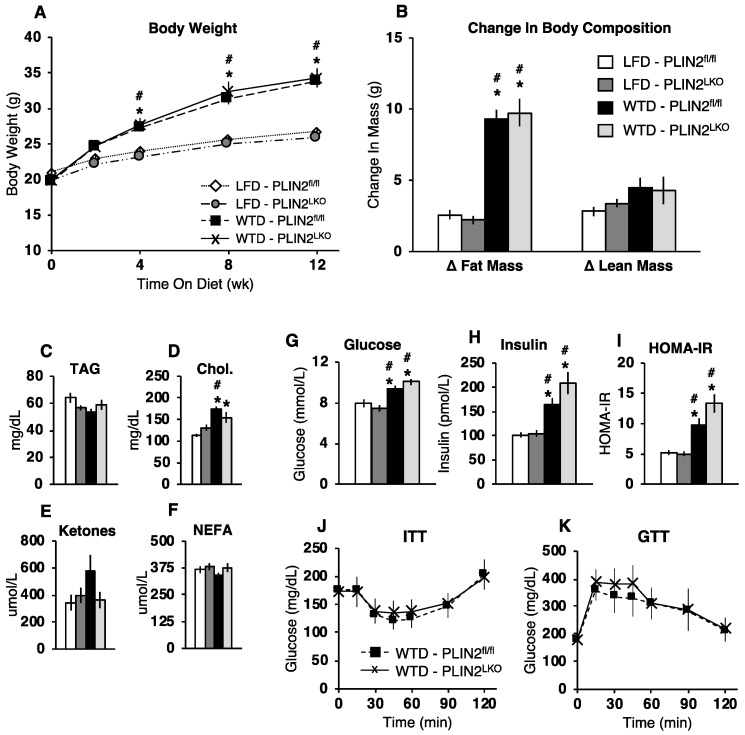
Hepatic PLIN2 loss does not affect body composition, plasma lipids, insulin–glucose homeostasis during LFD or WTD feeding. (**A**) Body weight over time during 12 weeks of LFD or WTD treatment. Animals began diet at 8 weeks of age. (**B**) Change in body composition from baseline, as determined by MRI, following 12 weeks of diet treatment. *n* = 8–13/group. (**C**) Serum triglyceride, (**D**) total cholesterol, (**E**) β -hydroxybutyrate (ketones), and (**F**) non-esterified fatty acids (NEFA) following a 6 h fast in 20-week-old mice treated with LFD or WTD for 12 weeks. *n* = 8–10/group. (**G**) 6 h fasting blood glucose. (**H**) plasma insulin, and (**I**) HOMA-IR in 20-week-old mice following 12 weeks of LFD or WTD. *n* = 10/group. (**J**) blood glucose response to an intraperitoneal injection of 0.75 IU/kg of insulin (insulin tolerance test, ITT) following a 6 h fast in mice fed LFD or WTD for 16 weeks beginning at 16 weeks of age. *n* = 8–10/group. (**K**) Blood glucose response to an intraperitoneal injection 1.5 mg/g of glucose (30% glucose in PBS, glucose tolerance test, GTT) following a 6 h fast in mice fed LFD or WTD for 18 weeks beginning at age 16 week of age. *n* = 5/group. Data are presented as the mean ± SEM. For **A**–**C**: * *p* ≤ 0.05 vs. LFD–PLIN2^fl/fl^ and # *p* ≤ 0.05 vs. LFD–PLIN2^LKO^, as determined by two-way ANOVA and Tukey’s post hoc analysis. For J and K, data were analyzed using students t-test.

We next investigated whether hepatic PLIN2 deficiency could alter the onset or severity of comorbidities associated with NAFLD including obesity, insulin resistance and dyslipidemia. In contrast to whole-body and antisense-oligonucleotide treatment models of PLIN2 deficiency [11,15,16,20], we observed no alterations in body weight or body composition between PLIN2^fl/fl^ and PLIN2^LKO^ during fed LFD or WTD for 12 weeks (Figure 2A,B). Fasting serum lipid metabolites including TAG, non-esterified fatty acids (NEFA), cholesterol, and ketones were also not different between genotypes within each diet treatment (Figure 2C–F). In addition, after 12 weeks of diet treatment, we observed no difference in fasting serum glucose, insulin and HOMA-IR between PLIN2^fl/fl^ and PLIN2^LKO^ during both LFD and WTD feeding (Figure 2G–I). Similarly, no differences in insulin sensitivity or glucose tolerance were observed between PLIN2^fl/fl^ and PLIN2^LKO^ during WTD feeding (Figure 2J,K). In sum, these data demonstrate that reduced hepatic PLIN2 does not protect against the development of HFD-induced weight and fat mass gain or obesity-associated alterations in insulin–glucose homeostasis early in the progression of NAFLD.

### 3.3. Liver-Specific Loss of PLIN2 Protects Against Hepatic Steatosis Following 12 Weeks of Western-Type Diet Feeding

**Figure 3:**
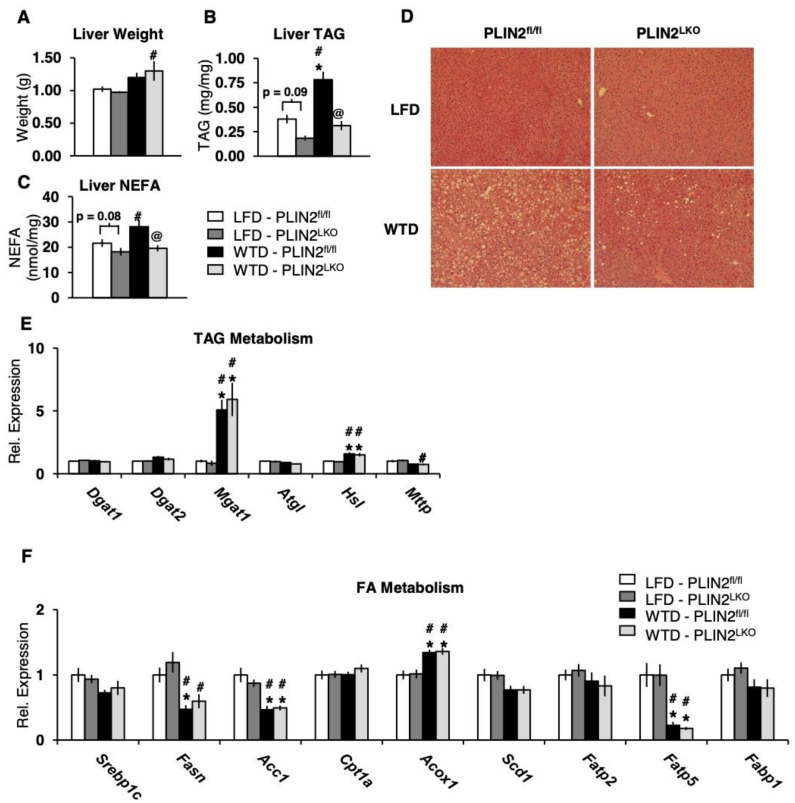
Ablation of hepatic PLIN2 protects mice against diet-induced hepatic steatosis in the absence of changes in lipogenic targets. (**A**) Liver weight, (**B**) liver TAG and (**C**) liver NEFA in 20-week-old mice after 12 weeks of LFD or WTD diet treatment. Liver weight *n* = 11–13/group. Liver TAG and NEFA, *n* = 6–8/grp. (**D**) Representative hematoxylin and eosin staining of liver sections harvested from 6 h fasted mice after 12 weeks of LFD or WTD treatment. (**E**) Gene expression of proteins involved in TAG metabolism and (**F**) FA metabolism in livers of 6 h fasted mice fed LFD or WTD for 12 weeks. *n* = 10–13/group. Data are presented as the mean ± SEM. * *p* ≤ 0.05 vs. LFD–PLIN2^fl/fl^, # *p* ≤ 0.05 vs. LFD–PLIN2^LKO^, and @ *p* ≤ 0.05 vs. WTD–PLIN2^fl/fl^, as determined by two-way ANOVA and Tukey’s post hoc analysis.

We next sought to delineate the impact of hepatic-specific deficiency of PLIN2 on lipid accumulation after 12 weeks of WTD feeding. Liver weights of WTD-fed mice were significantly elevated compared to LFD-fed mice. However, there was no significant effect of genotype on liver weight (Figure 3A). PLIN2 deficiency reduced liver TAG content by more than 50% following WTD feeding as compared to PLIN2^fl/fl^ controls (Figure 3B). In addition, there was a strong trend (*p* = 0.09) for reduced hepatic lipids in LFD fed PLIN2^LKO^ mice compared to LFD fed controls. Hepatic non-esterified fatty acids (NEFA) were reduced more than 30% in livers of WTD PLIN2^LKO^ mice as compared to WTD fed controls (Figure 3C). There was also a trend (*p* = 0.08) towards a reduction in NEFA content in PLIN2^LKO^ livers following LFD treatment. Consistent with the observed reduction in liver TAG content in WTD fed PLIN2^LKO^ mice, hematoxylin and eosin-stained liver sections from WTD-fed mice showed a marked reduction in lipid droplets in PLIN2^LKO^ mice compared with controls (Figure 3D). Furthermore, histological scoring revealed a decreased prevalence of grade 3 steatosis in WTD fed PLIN2^LKO^ livers compared to controls (Table S4). In summary, these findings are consistent with other studies investigating hepatic PLIN2 deficiency during high-fat feeding which demonstrated a reduction in liver TAG accumulation [11,14,15,16,19,20]. 

### 3.4. Hepatic PLIN2 Ablation Does Not Alter Expression of TAG and FA Genes

The TAG content of the liver represents the sum of multiple metabolic pathways: lipolysis and oxidation, TAG secretion in VLDL, as well as TAG and FA uptake. To explore which of these mechanisms may underlie protection against hepatic TAG accumulation in PLIN2^LKO^ mice, we analyzed the expression of genes involved in lipid metabolism in livers of mice fed LFD and WTD for 12 weeks. Expression of lipogenic genes *Dgat1*, *Dgat2*, *Mgat1*, *Scd1* as well as *Srebp1c* and its downstream genes, *Fasn* and *Acc1*, did not differ between genotypes within diet treatment groups (Figure 3E,F). Neither the mRNA expression of *Mttp*, an enzyme involved in export of TAG in VLDL, nor the expression of lipolytic enzymes *Atgl* and *Hsl* differed between genotypes within diet treatments. With regard to FA oxidative metabolism, mRNA expression of *Acox1* and *Cpt1a* did not differ between genotypes (Figure 3F). FA transporters *Fatp5, Fatp2* and *Fabp1* were also unchanged. In total, these results suggest that transcriptional modulation of genes involved in lipogenesis, lipolysis, lipid secretion and fatty acid uptake are not responsible for the observed reduction in hepatic TAG content in the livers of PLIN2^LKO^ mice.

### 3.5. The Absence of PLIN2 Increases Hepatocyte FA Oxidation in An Autophagic-Dependent Manner

When expressed on the surface of the LD, PLIN2 prevents association of ATGL and proteins involved in autophagy with the LD, thereby blocking their catabolic actions [22,23]. Given the absence of changes in lipogenic gene expression, we sought to determine whether autophagic-dependent lipolysis, termed lipophagy, is increased in livers of HFD fed PLIN2^LKO^ mice. 

**Figure 4:**
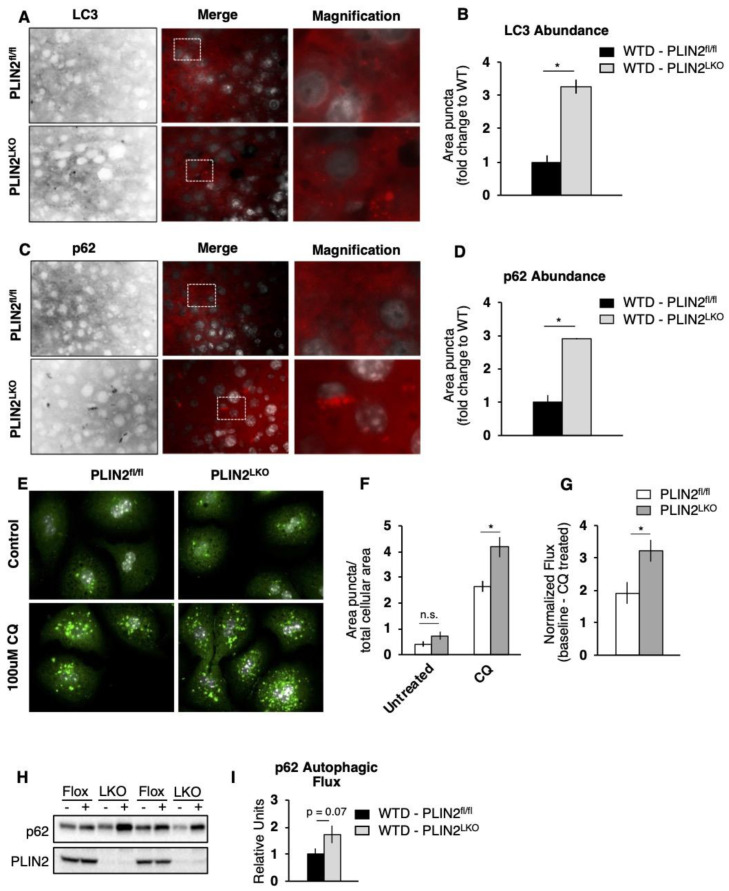
Liver-specific loss of PLIN2 enhances autophagic function. (**A**–**D**) Immunostaining of autophagic marker LC3 and p62 in frozen liver sections from PLIN2^fl/fl^ and PLIN2^LKO^ mice. Representative pictures and quantification of area occupied are shown. Higher magnification images in inverted black and white are shown for better visualization. *n* = 3. (**E**) Immunostaining and (**F**,**G**) quantitation of autophagic flux of the autophagosomal marker LC3 in fixed primary hepatocytes isolated from 8-month-old mice and incubated in M199 media in the presence or absence of 100 µM CQ for 6 h. (**H**) Representative blot and (**I**) densitometric quantitation of p62 autophagic flux from livers isolated from male mice fed HFD for 12 weeks and treated with (+) and without (–) autophagic inhibitors ex vivo. Flux is determined as the difference between treated and untreated p62 protein abundance and expressed relative to PLIN2^fl/fl^, *n* = 7. * *p* ≤ 0.05, as determined by one-way ANOVA and Tukey’s post hoc analysis.

We first performed immunohistochemical analysis of two different autophagic markers to visualize autophagic vesicles in liver sections. We observed significant increases in LC3- and p62-positive puncta in livers of PLIN2^LKO^ mice fed WTD (Figure 4A–D). Increased levels of these two markers suggest altered autophagic function in PLIN2^LKO^ liver, but do not indicate whether accumulation is due to accelerated biogenesis of autophagic vesicles (enhanced autophagy) or deficient clearance of those degradative vesicles (impaired autophagy). In order to elucidate between these two possibilities, we carried out functional analysis in primary hepatocytes and ex vivo analysis of autophagic function. PLIN2^LKO^ hepatocytes demonstrated a significant increase in the number of autophagosomes following treatment with chloroquine (CQ), which inhibits autophagic flux by decreasing autophagosome–lysosome fusion (Figure 4E,F). Furthermore, we observed a significant increase in LC3 flux, indicative of enhanced autophagic function, in PLIN2-deficient hepatocytes (Figure 4G). Consistent with the findings in primary cell culture, ex vivo analysis revealed a strong trend (*p* = 0.06) toward increased p62-dependent autophagic flux in the absence of hepatic PLIN2 (Figure 4H,I). In sum, using three different approaches we demonstrate that loss of hepatic PLIN2 enhances autophagic function in WTD-fed mice and suggest that accelerated lipophagy contributes to the reduced lipid accumulation observed in the liver of PLIN2^LKO^ mice.

### 3.6. Both Lipophagy and ATGL Contribute to FA Oxidation in PLIN2^LKO^ Hepatocytes

**Figure 5:**
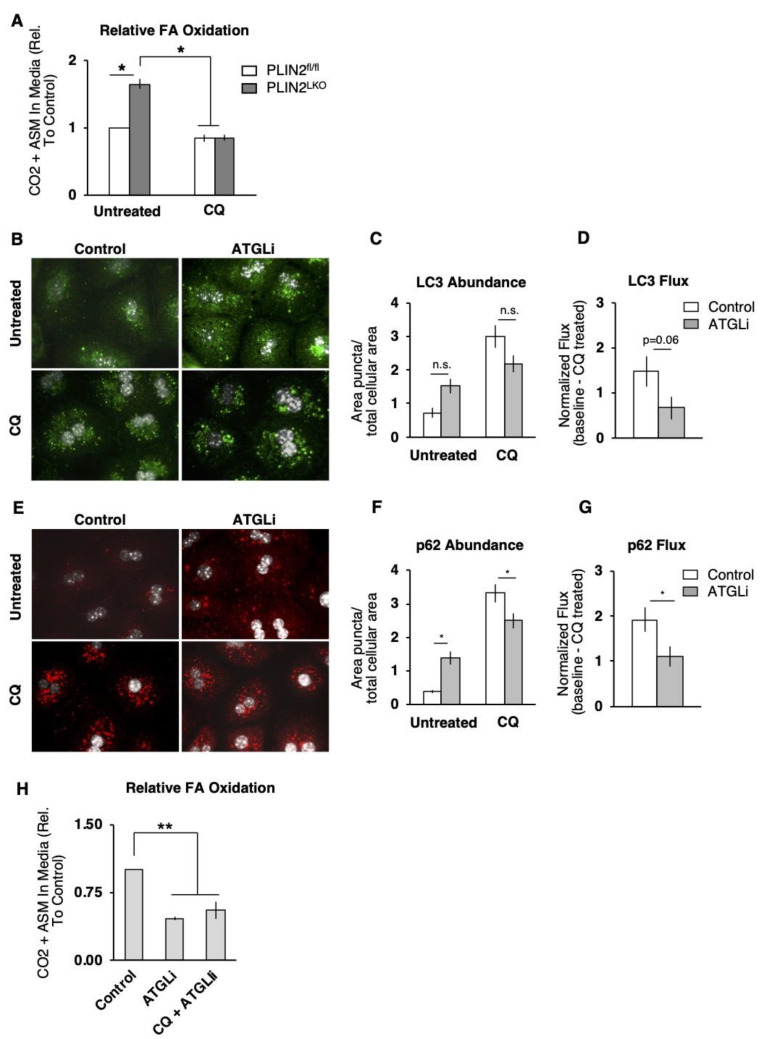
The integrated actions of lipophagy and ATGL increase FA oxidation in PLIN2-deficient hepatocytes. (**A**) ^14^C-labeled CO_2_ and acid-soluble metabolites in the media of primary hepatocytes isolated from 8-month-old mice incubated with 500 uM oleic acid (0.1 µCi/mL ^14^C-oleic acid) for 2 h and chased for 4 h in the presence of 100 µM chloroquine (CQ), *n* = 3 mice/group, expressed relative to PLIN2^fl/fl^. (**B**–**G**) Immunostaining of endogenous autophagosomal markers LC3 (**B**–**D**) and p62 (**E**–**G**) in fixed primary hepatocytes isolated from 12-week-old mice incubated for 6 h in serum free M199 media with and without 100 µM CQ. Quantification of puncta and flux was performed from 30 representative cells per treatment. Flux is expressed as the average increase in puncta following CQ treatment for each cell relative to the untreated group average. (**H**) ^14^C-labeled CO_2_ and acid-soluble metabolites in the media of primary hepatocytes isolated from 3-month-old PLIN2^LKO^ mice incubated with 500 µM oleic acid (0.1 µCi/mL ^14^C-oleic acid) for 2 h and chased for 4 h in the presence of 30 µM ATGListatin (ATGLi), or 30 µM ATGLi + 100 µM CQ. *n* = 3–4 mice/group, expressed relative to untreated. Data are presented as the mean ± SEM * *p* ≤ 0.05, ** *p* ≤ 0.01, as determined by (**A**–**G**) two-way ANOVA and (**H**) one-way ANOVA and Tukey’s post hoc analysis.

We next investigated whether increased rates of autophagy contribute to increased levels of FA oxidation in PLIN2^LKO^ hepatocytes. We observed a significant 65% increase in the rate of FA oxidation in isolated hepatocytes from chow fed PLIN2^LKO^ mice compared to hepatocytes from PLIN2^fl/fl^ mice (Figure 5A). Remarkably, increases in FA oxidation in PLIN2^LKO^ hepatocytes were completely blunted in the presence of the autophagy inhibitor CQ (Figure 5A) indicating that increased FA oxidation observed in PLIN2-deficient hepatocytes was due, in part, to increased rates of lipophagy.

While lipophagy and ATGL-mediated lipolysis were classically thought to be distinct processes, recent work has suggested that ATGL activity is required to increased rates of autophagy [24,25]. To investigate the contribution of ATGL to increased rates of autophagy in PLIN2^LKO^ hepatocytes, we measured the abundance of autophagic markers following inhibition of ATGL with ATGListatin (ATGLi). We hypothesized that blocking ATGLi activity would reduce autophagic function in PLIN2^LKO^ primary hepatocytes. In support of this hypothesis, we observed a significant decrease in p62-dependent autophagic flux (CQ puncta–untreated puncta) and a trend towards reduced LC3 flux (*p* = 0.06) following inhibition of ATGL in PLIN2^LKO^ hepatocytes (Figure 5B–G) These results indicate that in PLIN2-deficient hepatocytes ATGL activity enhances autophagic function.

Having established that hepatic PLIN2 deficiency (1) enhances autophagy and (2) increases FA oxidation in an autophagic-dependent manner and (3) that ATGL activity contributes to enhanced autophagy, we next sought to determine the relative contribution of ATGL to increased FA oxidation observed in the absence of PLIN2. Our analysis demonstrated that inhibition of ATGL by ATGLi treatment reduced FA oxidation significantly by 54% compared to untreated hepatocytes, confirming that ATGL activity contributes to FA oxidation in the absence of PLIN2 (Figure 5H). We hypothesized that the contribution of ATGL activity to increased FA oxidation in PLIN2^LKO^ hepatocytes is likely due to both ATGL-mediated regulation of autophagy and the autophagy-independent (lipolytic) actions of ATGL. To elucidate a possible autophagic-independent role of ATGL activity, we treated PLIN2^LKO^ hepatocytes with both CQ and ATGLi to simultaneously inhibit both autophagy and ATGL. We found that inhibition of ATGL and autophagy did not further reduce FA oxidation compared to treatment with ATGLi alone, arguing against an autophagy-independent role of ATGL activity in mediating FA oxidation in PLIN2^LKO^ hepatocytes, and suggesting that ATGL and autophagy act through an integrated pathway to increase FA oxidation following hepatic-specific PLIN2 ablation.

## 4. Discussion

NAFLD affects nearly 25% of the global population, with increasing prevalence among the elderly and individuals with obesity [8]. While clinically benign, NAFLD is a significant risk factor for the development of steatohepatitis and liver cancer [7]. Strategies to reduce hepatic TAG accumulation early in the course of NAFLD have the potential to prevent progression towards more serious forms of liver disease. Underlying the excessive accumulation of TAG in NAFLD is a dysregulation in the balance between lipid storage and breakdown. Breakdown of TAG within hepatic LD is mediated by the hydrolytic actions of ATGL and lipophagy. While the enzymatic machinery underlying lipolysis and lipophagy are different, they share a requirement for access to the LD surface for initiation. PLIN2, the most abundant PLIN isoform on LDs of steatotic livers, is believed to serve as a gatekeeper of LD access [1,21]. Separate reports have attributed reduced TAG following in the absence of intracellular PLIN2 to increased ATGL-mediated lipolysis [22] or lipophagy [23]. However, the relative contributions of these lipolytic pathways to PLIN2-mediated reductions in hepatic TAG has not been investigated. Here, we present data which elucidate the relative mechanistic roles of ATGL action, autophagy, and fatty acid oxidation in reducing hepatic LD TAG accumulation following hepatocyte PLIN2 deficiency during WTD feeding. Importantly, our studies provide new evidence that PLIN2 is a critical regulator of hepatic autophagy and ATGL-mediated lipolysis during WTD feeding. Using a mouse model of hepatic PLIN2 deficiency, we show that these two lipolytic pathways function in tandem to increase FA oxidation following PLIN2 loss and likely contribute to reduced accumulation of hepatic TAG.

Emerging data are redefining the interaction between ATGL-mediated lipolysis and lipophagy in the hydrolysis of hepatic LD TAG [34]. While traditionally thought of as distinct pathways, two recent studies challenge this schema and provide evidence that ATGL might function in parallel with lipophagy in hepatocytes [24,25]. Under this new model, ATGL preferentially targets to large LD, hydrolyzing TAG and reducing LD size. These small “LD remnants” are then targeted by lipophagy to complete LD TAG catabolism [25]. Our work supports this model in the context of hepatic PLIN2 deficiency, demonstrating that the combined inhibition of autophagy and ATGL activity did not have an additive effect to increase FA oxidation in PLIN2-deficient hepatocytes. These findings support the concept that lipolysis and lipophagy are not independent processes but work cooperatively.

In addition to PLIN2, another perilipin isoform, PLIN5 has been implicated in regulation of hepatic lipophagy and FA oxidation [31,32,33,35]. PLIN5 serves as a FA binding protein, delivering fatty acids released from lipolysis to the nucleus mediating FA activation of the transcription factor SIRT1, a master regulator of autophagy. In hepatocytes, PLIN5 ablation reduces FA oxidation and impairs autophagy [33,35]. Interestingly, we observed a small, but significant increase in PLIN5 protein abundance following the absence of PLIN2. Although the mechanisms underlaying the upregulation of PLIN5 are unclear, this observation suggests that transcriptional regulation of SIRT1 through PLIN5 might contribute to the increased autophagy we observed in PLIN2-deficient livers. 

While our study demonstrated a role for increased TAG hydrolysis in mediating the reduced hepatic TAG accumulation observed with PLIN2 deficiency, another study investigating a more chronic, 30-week WTD treatment attributed the reduced hepatic TAG in hepatic-specific PLIN2-deficient mice to reduced lipogenesis as evidenced by reduced mRNA expression of the lipogenic transcription factor *Srebp1c* and its downstream genes *Fasn* and *Acc*. In contrast to this work, we did not observe changes in lipogenic gene expression nor in the expression of FA transporters, arguing against a contribution from reduced de novo lipogenesis to the reductions in TAG observed in PLIN2-deficient livers, at least early in the progression of NAFLD. The differences between these studies may be explained by the progression and severity of NAFLD at the time of study. Following 30 weeks of WTD feeding, mice develop significant increases in fibrosis and inflammation, while in our study, we observed no changes in hepatic inflammation or fibrosis after 12 weeks of WTD feeding (Figure S1 and Table S4) [19]. In total, these results suggest that liver-specific deficiency of PLIN2 may act through distinct mechanisms to reduce hepatic TAG depending on the stage and severity of diet-induced NAFLD progression. Additional functional studies will be necessary to understand how changes in FA oxidation, lipogenesis, as well as FA uptake and secretion, contribute to reductions in hepatic TAG following PLIN2 loss during NAFLD progression. 

Hepatic steatosis is strongly correlated with obesity and diabetes. However, the effect of PLIN2 loss on these comorbidities has been inconsistent across mouse models of PLIN2 deficiency. These inconsistencies have recently been reconciled in studies using a chronic, 30-week WTD model of NAFLD which suggested that PLIN2 in extra-hepatic tissues such as muscle and adipose mediates metabolic impairments associated with NAFLD, while hepatic PLIN2 specifically contributes to reduced hepatic steatosis, fibrosis, and immune cell infiltration [19]. Our studies build upon this work, demonstrating that after 12 weeks of WTD feeding, deficiency of PLIN2 in hepatocytes fails to protect against the development of obesity and insulin resistance. In total, studies of hepatocyte-specific PLIN2 deficiency suggest that hepatic PLIN2 does not affect the onset or severity of common metabolic complications associated with NAFLD at any stage of the disease progression. Furthermore, these studies imply that targeted loss of PLIN2 in one or more extra-hepatic tissues such as muscle, adipose, or brain may have therapeutic potential in the treatment of obesity associated weight gain and insulin resistance. 

In summary, our data demonstrate that hepatic ablation of PLIN2 increases cellular FA oxidation through cooperation between autophagy and ATGL-mediated lipolysis. Additionally, we demonstrate that hepatic loss of PLIN2 protects mice from diet-induced hepatic steatosis early in the progression of NAFLD but is not associated with reductions in adiposity and insulin resistance. Overall, these experiments provide new insight into the mechanisms by which the absence of hepatic PLIN2 reduces hepatic TAG accumulation.

## Supplementary Materials

The following are available online at https://www.mdpi.com/article/10.3390/cells10051016/s1, Figure S1: Hepatic-specific deficiency of PLIN2 does not alter expression of inflammatory genes in liver after 12 weeks of WTD. Table S1: Nutrient and ingredient composition of experimental diets as compared to common high-fat diets; Table S2: Fatty acid composition of experimental diets as compared to common high-fat diets; Table S3: Primer sequences used in qPCR analysis; Table S4: Reduced prevalence of Grade 3 steatosis in Western-type diet-fed PLIN2^LKO^ livers.
