# Supplementary material for: Integrated Action of Autophagy and Adipose Tissue Triglyceride Lipase Ameliorates Diet-Induced Hepatic Steatosis in Liver-Specific PLIN2 Knockout Mice
Source: Cells. 2021 Apr 25;10(5):1016. doi: 10.3390/cells10051016 (PMC8145136; doi:10.3390/cells10051016)
Supplement: Supplementary file 1 [file cells-10-01016-s001.zip › Figure S1.pdf]

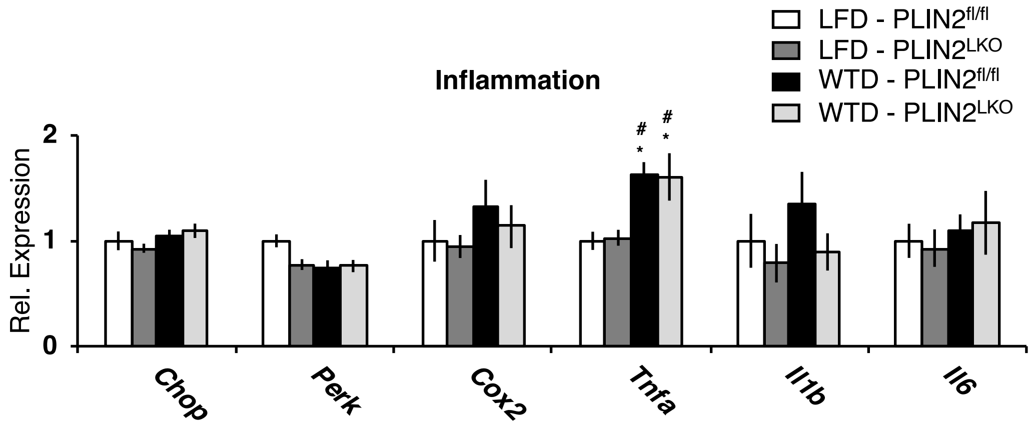


**Figure S1. Hepatic specific deficiency of PLIN2 does not alter expression of inflammatory genes in liver after 12 weeks of western type diet feeding.** Gene expression as measured by qPCR in livers of 6 hr fasted mice fed low fat (LFD) or western-type (WTD) diet treatment for 12 weeks. n=10-13/group. Data are presented as mean ± SEM. *p ≤ 0.05 vs LFD–PLIN2$^{fl/fl}$, #p ≤ 0.05vs LFD–PLIN2$^{LKO}$, @ p≤ 0.05 vs WTD–PLIN2$^{fl/fl}$ as determined by two-way ANOVA and Tukey's post-hoc analysis.
