# Supplementary material for: Integrated Action of Autophagy and Adipose Tissue Triglyceride Lipase Ameliorates Diet-Induced Hepatic Steatosis in Liver-Specific PLIN2 Knockout Mice
Source: Cells. 2021 Apr 25;10(5):1016. doi: 10.3390/cells10051016 (PMC8145136; doi:10.3390/cells10051016)
Supplement: Supplementary file 1 [file cells-10-01016-s001.zip › Table S1.pdf]

Macronutrient Composition (g/kg)

	45% HFD	60% HFD	WTD	LFD
Protein	24.00	26.20	20.10	16.30
Fat	24.00	34.90	23.70	4.20
Carbohydrate	41.00	26.30	45.90	71.10
Cholesterol (ppm)*	192.00	275.00	593.00	97.00
Fiber	50.00	50.00	50.00	50.00

Ingredients (g/kg)

	45% HFD	60% HFD	WTD	LFD
Sucrose	172.80	68.80	340.00	0.00
Corn Starch	72.80	0.00	56.86	701.00
Maltodextrin	100.00	500.00	60.00	150.00
Casein - Vitamin Tested	200.00	200.00	195.00	195.00
Milk Fat	x	x	210.00	30.00
Lard	177.50	245.00	x	x
Soybean Oil	25.00	25.00	20.00	20.00
Corn Oil	x	x	0.00	0.00
Powdered Cellulose	50.00	50.00	50.00	50.00
AIN-93G Mineral Mix/Fiber	x	x	35.00	35.00
AIN-93 Vitamin Mix/ Fiber	x	x	10.00	10.00
Calcium Carbonate	5.50	5.50	0.00	0.00
DL-Methionine	x	x	0.00	0.00
L-cysteine	3.00	3.00	3.00	3.00
Choline Bitartate	2.00	2.00	3.00	3.00
Ethoxyquin	x	x	0.00	0.00
t-Butylhydroquinone	x	x	0.04	0.04

Macronutrient Composition (%kcal)

	45% HFD	60% HFD	WTD	LFD
Protein	20.00	20.00	17.00	17.00
Fat	45.00	60.00	39.00	10.00
Carbohydrate	35.00	20.00	44.00	73.00

Table S1. Nutrient and ingredient composition of experimental diets as compared to common high fat diets. All diets formulated by Research Diets, 45% high fat diet (HFD), D12451; 60% HFD, D12492, western-type diet (WTD), D15102305; low fat diet (LFD), D15102305.
