# Supplementary material for: Integrated Action of Autophagy and Adipose Tissue Triglyceride Lipase Ameliorates Diet-Induced Hepatic Steatosis in Liver-Specific PLIN2 Knockout Mice
Source: Cells. 2021 Apr 25;10(5):1016. doi: 10.3390/cells10051016 (PMC8145136; doi:10.3390/cells10051016)
Supplement: Supplementary file 1 [file cells-10-01016-s001.zip › Table S2.pdf]

Name	Lipid Number	45% HFD	60% HFD	WTD	LFD
Butyric Acid	C4	0.00	0.00	1.42	0.21
Caproic Acid	C6	0.00	0.00	0.84	0.13
Caprylic Acid	C8	0.00	0.00	0.49	0.07
Capric Acid	C10	0.00	0.00	1.11	0.17
Lauric Acid	C12	0.03	0.04	1.24	0.19
Myristic Acid	C14	0.48	0.66	4.44	0.67
Myristoleic Acid	C14:1	0.00	0.00	0.67	0.10
Pentadecylic Acid	C15	0.00	0.00	0.00	0.00
Palmitic Acid	C16	8.78	11.91	12.08	2.20
Palmitoleic Acid	C16:1	0.64	0.88	1.02	0.16
Heptadecanoic Acid	C17	0.12	0.17	0.00	0.00
Octadecanoic Acid	C18	4.70	6.41	5.54	0.98
Oleatic Acid	C18:1	15.12	20.38	12.16	2.69
Linoleic Acid	C18:2	10.88	13.93	3.32	2.45
a-Linolenic Acid	C18:3	0.89	1.08	0.95	0.42
Stearidonic Acid	C18:4	0.00	0.00	0.00	0.00
Eicosanoic Acid	C20	0.10	0.13	0.44	0.08
Gondoic Acid	C20:1	0.27	0.37	0.01	0.01
Eicosadienoic Acid	C20:2	0.31	0.42	0.00	0.00
Dihomo-y-linolenic Acid	C20:3	0.10	0.14	0.00	0.00
Aracidonic Acid	C20:4	0.10	0.14	0.00	0.00
Eicosapentaenoic Acid (EPA)	C20:5	0.00	0.00	0.00	0.00
Heneicosapentaenoic Acid	C21:5	0.00	0.00	0.00	0.00
Docosanoic Acid	C22	0.01	0.01	0.01	0.01
Docosatetraenoic Acid	C22:4	0.00	0.00	0.00	0.00
Docosapentaenoic Acid	C22:5	0.03	0.04	0.00	0.00
Decosahexaenoic Acid (DHA)	C22:6	0.00	0.00	0.00	0.00
Tetracosanoic Acid	C24	0.01	0.01	0.01	0.01

	45% HFD	60% HFD	WTD	LFD
Saturated Fat (% Total Fat)	33.44	34.09	60.38	44.7
MUFA (% Total Fat)	37.63	38.13	30.29	28.07
PUFA (% Total Fat)	28.93	27.78	9.32	27.23
Saturated Fat (% Total Energy)	14.24	19.33	23.91	4.08
MUFA (% Total Energy)	16.02	21.62	12.00	2.56
PUFA (% Total Energy)	12.32	15.75	3.69	2.49
Total Fat (% Total Energy)	42.58	56.71	39.61	9.13

Table S2. Fatty acid composition of experimental diets as compared to common high fat diets. All diets formulated by Research Diets, 45% high fat diet (HFD), D12451; 60% HFD, D12492, western-type diet (WTD), D15102305; low fat diet (LFD), D15102305.
