# Supplementary material for: Integrated Action of Autophagy and Adipose Tissue Triglyceride Lipase Ameliorates Diet-Induced Hepatic Steatosis in Liver-Specific PLIN2 Knockout Mice
Source: Cells. 2021 Apr 25;10(5):1016. doi: 10.3390/cells10051016 (PMC8145136; doi:10.3390/cells10051016)
Supplement: Supplementary file 1 [file cells-10-01016-s001.zip › Table S3.pdf]

| Name | Forward Sequence | Reverse Sequence |
|---|---|---|
| ACC1 | CATCACCATCAGCCTGGTTACA | ACTGTGTACGCTCTTCGGCAT |
| ACOX1 | ACGCCACTTCCTTGCTCTTC | AGATTGGTAGAAATTGCTGCAAAA |
| ATGL | TGTGGCCTCATTCCTCCTAC | TCGTGGATGTTGGTGGAGCT |
| Cideb | CATCACAGACACGGAAGGCTC | CAATGGCCTGCTAAGGTCAGT |
| COX2 | CACAGCCTACCAAAACAGCCA | GCTCAGTTGAACGCCTTTTGA |
| CPT1a | AGTGGCCTCACAGACTCC | GCCCATGTTGTACAGCTTCC |
| DGAT1 | TCACCACACACCAATTCAGG | GACGGCTACTGGGATCTGA |
| DGAT2 | CGCAGCGAAAACAAGAATAA | GAAGATGTCTTGGAGGGCTG |
| FASN | GCTGCGGAAACTTCAGGAAAT | AGAGACGTGTCACTCCTGGACTT |
| FSP-27 | ATTGTGCCATCTTCCTCCAG | GTCGTGTTAGCACCGCAGAT |
| HSL | GGAGCACTACAAACGCAACGA | TCGGCCACCGGTAAAGAG |
| IL-1B | CTGGTGTGTGACGTTCCCATT | CCGACAGCACGAGGCTTT |
| IL-6 | CCCCAATTTCCAATGCTCTCC | CGCACTAGGTTTGCCGAGTA |
| Map1lc3a | GACCGCTGTAAGGAGGTGC | CTTGACCAACTCGCTCATGTTA |
| Map1lc3b | TTATAGAGCGATACAAGGGGGAG | CGCCGTCTGATTATCTTGATGAG |
| MGAT1 | GAGTAACGGGCCGGTTTCA | AGACATTGCCACCTCCATCCT |
| Mttp | AGTGCAGTTCTCACAGTACCCGTT | AGCATATCGTTCTGGTGGAAGGGA |
| PLIN1 | CAACAGCACCAAAGAAGCCC | GCACCCTGTACACCCTTCTC |
| PLIN2 | CAATTTGTGGCTCCAGCTTC | CCCGTATTTGAGATCCGTGT |
| PLIN3 | TGGTGATTAGCGGAGTGGAC | GTAGTTCTGCTCCTGTCGCT |
| PLIN4 | GCATCTTCACTGCTGGTCAC | CTGCCCCCTCATCTAAAGTG |
| PLIN5 | CCGTGTCCAGTGCTTACAAC | AGGGCAGCTTCTCTTCCAAT |
| SCD1 | CCGGAGACCCCTTAGATCGA | TAGCCTGTAAAAGATTTCTGCAAACC |
| Sqstm1 | AGGATGGGGACTTGGTTGC | TCACAGATCACATTGGGGTGC |
| SREBP1c | GGAGGGGTAGGGCCAACGGCCT | CATGTCTTCGAAAGTGCAATCC |
| TNF-a | ATGGGCTTTCCGAATTCAA | GAGGCAACCTGACCACTCTC |

**Table S3.** Primer Sequences
