# Supplementary material for: Integrated Action of Autophagy and Adipose Tissue Triglyceride Lipase Ameliorates Diet-Induced Hepatic Steatosis in Liver-Specific PLIN2 Knockout Mice
Source: Cells. 2021 Apr 25;10(5):1016. doi: 10.3390/cells10051016 (PMC8145136; doi:10.3390/cells10051016)
Supplement: Supplementary file 1 [file cells-10-01016-s001.zip › Table S4.pdf]

| | Steatosis Grade | | | | |
|---|---|---|---|---|---|
| Group | **0** | **1** | **2** | **3** | **NASH** |
| LFD - PLIN2$^{fl/fl}$ | 0 | 67% (4) | 33% (2) | 0 | 0 |
| LFD - PLIN2$^{LKO}$ | 0 | 83% (5) | 17% (1) | 0 | 0 |
| WTD - PLIN2$^{fl/fl}$ | 0 | 0 | 33% (2) | 67% (4) | 0 |
| WTD - PLIN2$^{LKO}$ | 0 | 17% (1) | 50% (3) | 33% (2) | 0 |

**Table S4. Reduced prevalence of Grade 3 steatosis in western-type diet fed PLIN2$^{LKO}$ livers.** Histopathological scoring of the degree of steatosis in liver sections collected from mice from 12 weeks of LFD or WTD feeding n=6. Grades represent the percentage of the liver section that was occupied by fat at 100 $\times$ magnification in 20 fields. Grade 0 = < 5% view; Grade 1 = 5 to 25%; Grade 2 = 26 to 50%; Grade 3 = 51 to 75%; Grade 4 = 76% to 100%. Inflammatory foci and ballooning degeneration of hepatocytes were also examined with prevalence reported under NASH column. Data are presented as the percentage of animals from the sample group presenting with a given steatosis grade with number of animals shown in parentheses.
